# Herpes Simplex Virus Type 1 Single Strand DNA Binding Protein and Helicase/Primase Complex Disable Cellular ATR Signaling

**DOI:** 10.1371/journal.ppat.1003652

**Published:** 2013-10-03

**Authors:** Kareem N. Mohni, Samantha Smith, Alexander R. Dee, April J. Schumacher, Sandra K. Weller

**Affiliations:** Department of Molecular, Microbial and Structural Biology and the Molecular Biology and Biochemistry Graduate Program, University of Connecticut Health Center, Farmington, Connecticut, United States of America; University of North Carolina at Chapel Hill, United States of America

## Abstract

Herpes Simplex Virus type 1 (HSV-1) has evolved to disable the cellular DNA damage response kinase, ATR. We have previously shown that HSV-1-infected cells are unable to phosphorylate the ATR substrate Chk1, even under conditions in which replication forks are stalled. Here we report that the HSV-1 single stranded DNA binding protein (ICP8), and the helicase/primase complex (UL8/UL5/UL52) form a nuclear complex in transfected cells that is necessary and sufficient to disable ATR signaling. This complex localizes to sites of DNA damage and colocalizes with ATR/ATRIP and RPA, but under these conditions, the Rad9-Rad1-Hus1 checkpoint clamp (9-1-1) do not. ATR is generally activated by substrates that contain ssDNA adjacent to dsDNA, and previous work from our laboratory has shown that ICP8 and helicase/primase also recognize this substrate. We suggest that these four viral proteins prevent ATR activation by binding to the DNA substrate and obstructing loading of the 9-1-1 checkpoint clamp. Exclusion of 9-1-1 prevents recruitment of TopBP1, the ATR kinase activator, and thus effectively disables ATR signaling. These data provide the first example of viral DNA replication proteins obscuring access to a DNA substrate that would normally trigger a DNA damage response and checkpoint signaling. This unusual mechanism used by HSV suggests that it may be possible to inhibit ATR signaling by preventing recruitment of the 9-1-1 clamp and TopBP1.

## Author Summary

DNA viruses that replicate in the nucleus have been shown to both activate and inactivate various components of the cellular DNA damage response (DDR). Previous reports from our laboratory and others have demonstrated that Herpes Simplex Virus (HSV) utilizes some aspects of the DDR while inactivating others. Paradoxically, HSV utilizes the DDR kinase ATR to complete its life cycle while at the same time disabling the kinase from activating DDR signaling. In this report we provide detail describing the mechanism of ATR inactivation. ATR is normally activated in response to single strand DNA (ssDNA), which serves as a scaffold to recruit several proteins required for complete ATR activation. In this paper we provide evidence that the HSV encoded ssDNA binding protein and helicase/primase complex function to mask the DNA substrate that recruits the ATR kinase activator. This represents the first example of viral DNA replication proteins masking a DNA substrate that could be sensed by the cell as damaged DNA and activate checkpoint signaling. It also explains how ATR can be recruited to sites of viral DNA replication in the absence of checkpoint signaling.

## Introduction

**Figure 1:**
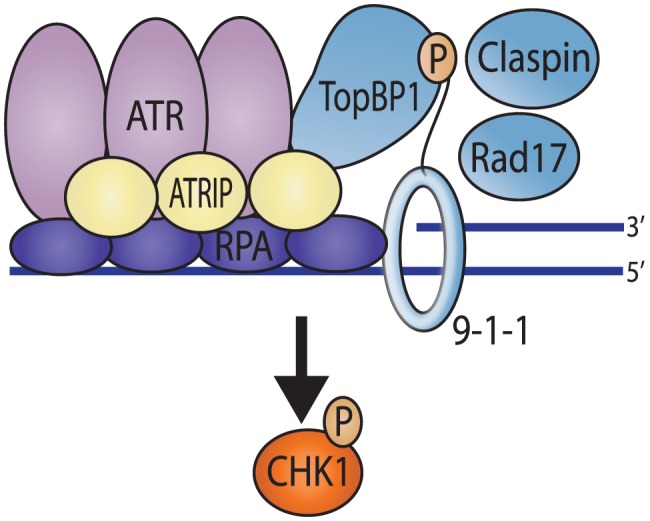
Recruitment of ATR pathway proteins to sites of DNA damage in uninfected cells. The ssDNA at sites of damage is coated by Replication protein A (RPA) and recruits ATR through a direct interaction with the ATR interacting protein (ATRIP). This RPA coated ssDNA also promotes the loading of the 9-1-1 (Rad9-Rad1-Hus1) checkpoint clamp by Rad17 onto the junction of the ss/dsDNA. TopBP1 is recruited via an interaction with the phosphorylated C-terminal tail of Rad9. TopBP1 then activates ATR kinase activity resulting in phosphorylation of Chk1, which is promoted by Claspin.

Eukaryotic cells have evolved a complex set of pathways to repair DNA and ensure the faithful duplication of the genome [1]–[4]. The cellular DNA damage response is orchestrated by the phosphoinositide 3-kinase-related kinases DNA-PK (DNA-dependent protein kinase), ATM (ataxia-telangiectasia-mutated) and ATR (ATM and Rad3 related). DNA-PK and ATM are activated in response to DNA double strand breaks (DSBs), and ATR is activated in response to substrates which contain single stranded DNA (ssDNA) adjacent to double stranded DNA (dsDNA) such as the DNA found at stalled replications forks. An ATR-activating structure is also produced by resection of DSBs in an ATM-dependent manner; thus, if resection occurs, ATM activation generally results in ATR activation as well. The ssDNA at sites of damage is coated by Replication protein A (RPA) and recruits ATR through a direct interaction with the ATR interacting protein (ATRIP) [5]–[7]. ATR signaling also requires the localization of the 9-1-1 (Rad9-Rad1-Hus1) checkpoint clamp to sites of DNA damage [8]–[10]. A major function of the 9-1-1 clamp is to recruit the ATR kinase activator, TopBP1 [11], which promotes phosphorylation of ATR-specific substrates such as serine345 on Chk1 (Checkpoint kinase 1) and serine33 on RPA [12], [13] (Summarized in Fig. 1).

Herpes Simplex Virus type 1 (HSV-1) is a double-stranded DNA virus that replicates in the nucleus of the host cell and as such must contend with the cellular DNA damage response [14]. DNA-PK, a key component of the classical nonhomologous end-joining (C-NHEJ) pathway, is degraded by the viral encoded ubiquitin ligase, ICP0, in some cell types. This degradation likely results in the inactivation of C-NHEJ, at least in cells in which DNA PK is degraded [15]–[17]. In addition, we have previously reported that HSV-1 infection disables ATR activation [7], [18] a surprising observation given that HSV-1 DNA replication activates the ATM signaling pathway [17], [19], [20]. In HSV-1-infected cells, ATR phosphorylation of RPA and Chk1 is inhibited even in the presence of replicative stress [7]; however, ATR/ATRIP and RPA are recruited to viral replication compartments, where they play positive roles during infection [7], [17]. Furthermore, we have recently shown that all of the ATR pathway proteins are recruited to viral replication compartments and that ATRIP, RPA, TopBP1, and CINP are required for efficient HSV-1 replication [18]. Thus, it appears that although HSV-1 commandeers ATR pathway proteins, it has evolved to manipulate the host DNA damage response by inactivating DNA-PK and ATR signaling.

HSV-1 encodes seven essential replication proteins: an origin binding protein, UL9, a single-stranded DNA binding protein, ICP8, a three subunit helicase/primase complex (UL8/UL5/UL52), a polymerase, UL30, and a polymerase accessory factor, UL42 [21], [22]. ICP8 is the nucleating factor of replication compartment formation, and no detectable intra-nuclear structures are formed in its absence [23], [24]. The helicase/primase complex is a heterotrimer consisting of UL8, UL5, and UL52 subunits. All of the catalytic properties of the complex are retained in a subcomplex consisting of UL5 and UL52 [25], while UL8 appears to be important for the nuclear import of UL5 and UL52 [26], [27]. UL8 interacts with other replication proteins including ICP8, UL9 and UL30 and may also mediate protein-protein interactions at a replication fork [28]–[30]. For instance, UL8 is required for ICP8 to stimulate helicase/primase activity [31]–[36].

Here we present evidence that HSV-1 can inhibit ATR signaling by preventing essential ATR pathway proteins from accessing sites of DNA damage. We show that inhibition of ATR signaling during infection is time-dependent and requires the HSV-1 replication proteins ICP8 and helicase/primase. In cells transfected with plasmids encoding ICP8 and helicase/primase, a nuclear complex is formed that we have called the four-protein complex. This complex localizes to sites of DNA damage and recruits ATR/ATRIP and RPA while excluding Rad9 and TopBP1. We propose that the presence of viral proteins at the sites of DNA damage compete for the loading signals for the 9-1-1 complex. The failure to recruit Rad9 and TopBP1 to sites of damage likely explains the lack of ATR signaling during infection.

## Results

### ICP8 and UL8 are required to inhibit ATR signaling during HSV-1 infection

**Figure 2:**
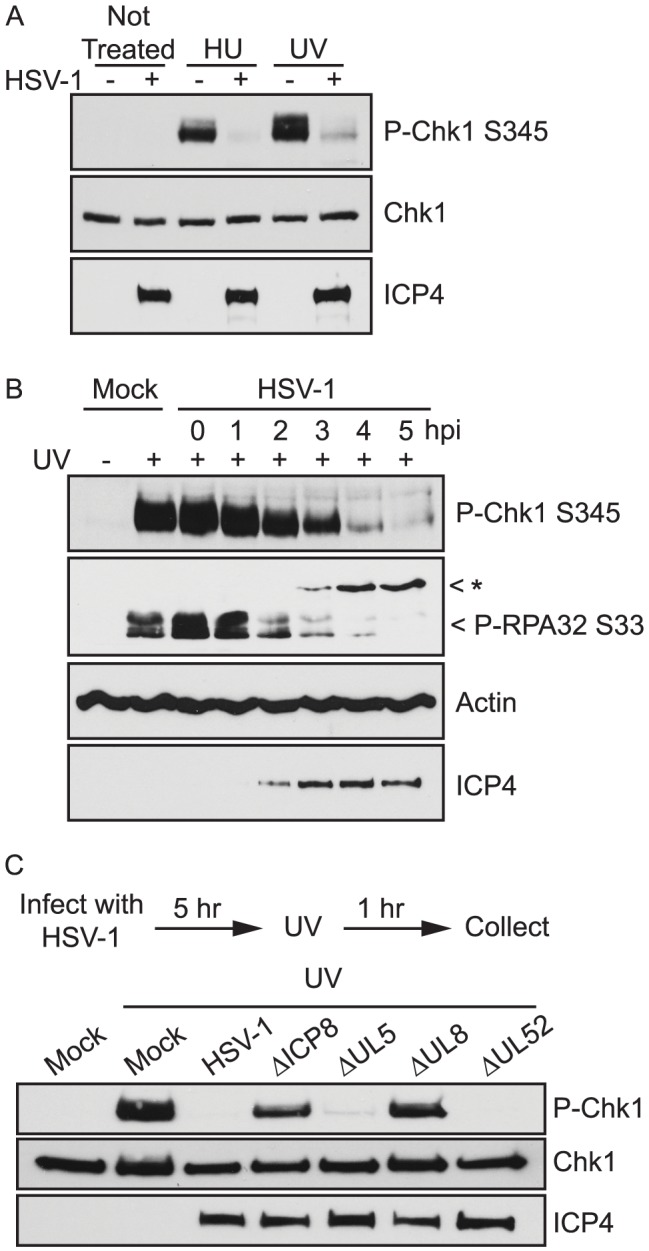
HSV-1 inhibition of ATR signaling requires ICP8 and UL8. (A) Vero cells were either mock-infected or infected with HSV-1 at an MOI of 10 PFU/cell. At 5 hours post infection cells were treated with HU for 1 hour or UV and allowed to recover for 1 hour. (B) Vero cells were either mock-infected or infected with HSV-1 at an MOI of 10 PFU/cell. Cells were treated with UV at the indicated time post infection and allowed to recover for 1 hour. (C) Vero cells were either mock-infected or infected with the indicated HSV-1 mutant viruses at an MOI of 10 PFU/cell. At 5 hours post infection cells were treated with UV and allowed to recover for one hour. All cell lysates were analyzed by Western blot with the indicated antibodies. The band marked with an asterisk (*) in the P-RPA-S33 blot corresponds to a non-specific band that does not cross react with antibodies to endogenous RPA and likely represents cross-reactivity with a viral protein.

We have previously shown that ATR is inhibited during infection even in the presence of hydroxyurea (HU) [7] which is known to stall cellular as well as viral replication forks [37]. This observation suggests that ATR cannot sense or respond to stalled forks in HSV-infected cells, and we initiated the current study to identify the viral mechanisms responsible for ATR inhibition. To test if this inhibition was specific to stalled replication forks or can be generalized to other forms of damage known to activate ATR, we treated infected cells with UV, which stalls replication forks and, in addition, creates cross-linked bases that need to be repaired by nucleotide excision repair. Figure 2A shows that HU- and UV-treatment of uninfected cells induced the phosphorylation of Chk1; however, in HSV-1-infected cells, no detectable phosphorylated Chk1 was observed, even after treatment with HU or UV. These data indicate that ATR signaling is inhibited during HSV-1 infection.

To determine how soon after infection ATR signaling is disabled, we damaged cells with UV at various times post infection and monitored the phosphorylation of Chk1 and RPA (Fig. 2B). ATR-mediated phosphorylation of Chk1 S345 and RPA32 S33 was detected at 1 and 2 hours post infection, began to decline at 3 and 4 hours post infection, and was greatly reduced by 5 hours post infection. The timing of the inhibition coincides with early events in the virus life cycle and implicates an early gene product or DNA replication itself as the viral factor responsible for ATR inhibition.

After repair, RPA is dephosphorylated by phosphatases in order to clear the damage signal. It is possible that, rather than inhibit ATR signaling, HSV-1 potentiates phosphatase activity, thus making ATR-dependent phosphorylation events appear diminished in infected cells. To rule out activation of a phosphatase, we damaged cells and compared the time it took for the damage signal (RPA phosphorylation) to be removed in mock and infected cells. We observed that RPA S33 became dephosphorylated with the same kinetics in mock infected and HSV-1 infected cells (Fig. S1). When cells were damaged prior to infection, the phosphorylation marks persisted up to 6 hours post infection in both mock- and HSV-1-infected cells. On the other hand, in figure 2B, ATR signaling was inhibited when cells were damaged as early as 4 h.p.i. If HSV were activating a phosphatase, we would expect to see the phosphorylation marks in Figure S1 dissipate at the same time point that HSV prevents ATR signaling in response to new damage, and this is not the case. Thus, it appears that HSV-1 prevents the phosphorylation of these sites rather than potentiating dephosphorylation.

To identify the viral proteins responsible for ATR inhibition we infected cells with a panel of viruses defective in both immediate early (IE) and early (E) genes. Infected cells were damaged with HU at 5 hours post infection, and the phosphorylation of Chk1 was monitored by Western blot (Table 1). Mutants defective in the IE proteins ICP0 and ICP22 were able to inhibit ATR signaling while mutants defective in ICP4 and ICP27 were not. ICP0 and ICP22 are non-essential in cell culture, and at the high multiplicity of infection (MOI) used in this study, null-mutants are able to progress to E gene expression and DNA replication. ICP4 and ICP27 are essential in cell culture, and in the absence of these proteins infected cells are unable to carry out E gene expression or DNA replication. To test if E gene expression or DNA replication was required to inhibit ATR signaling, we infected cells with mutants defective in early replication proteins or in the presence of replication inhibitors. Mutants defective in UL5, UL52, and UL30, were able to inhibit ATR signaling (Table 1 and Figure 2C). Signaling was also inhibited during infection in the presence of both helicase/primase inhibitors and polymerase inhibitors (Table 1). These data suggest that DNA replication *per se* is not required to inhibit ATR signaling. On the other hand, viral mutants deficient in ICP8 and UL8 failed to inhibit ATR signaling in HeLa and Vero cells (Table 1 and Fig. 2C). Together these data strongly implicate ICP8 and UL8 as the viral proteins responsible for inhibiting ATR signaling.

**Table 1:** Summary of HSV-1 mutants ability to disable ATR signaling.

	P-CHK1 S345	ATR inhibition
HSV-1	No	Yes
Immediate Early Mutants		
ΔICP0	No	Yes
ΔICP4	Yes	No
ΔICP22	No	Yes
ΔICP27	Yes	No
ΔICP4/ICP27	Yes	No
ΔICP4/22/27/47	Yes	No
Replication Mutants		
ΔICP8	Yes	No
ΔUL5	No	Yes
ΔUL8	Yes	No
ΔUL30	No	Yes
ΔUL52	No	Yes
Replication Inhibitors		
HSV-1+BAY	No	Yes
HSV-1+PAA	No	Yes

HeLa cells were infected with the indicated HSV-1 mutants at an MOI of 10 PFU/cell. At 5 hours post infection cells were treated with HU for 2 hours. All cell lysates were analyzed by Western blot for P-Chk1 S345, total Chk1, and ICP4/ICP8 as described in the legend to Figure 2. The ΔUL30 mutant was used at an MOI of 2 PFU/cell. The helicase/primase inhibitor, BAY 57-1293 (BAY), and the polymerase inhibitor, phosphonoacetic acid (PAA), were used as described in Materials and Methods.

### ICP8 and UL8 are sufficient to inhibit ATR signaling

HSV-1 replication proteins such as ICP8 have been implicated in reorganization of the infected cell nucleus resulting in the formation of replication compartments [38], [39]. Replication compartment formation occurs through an ordered assembly of replication proteins [23], [24], [40], [41]. By adding helicase/primase inhibitors to block DNA replication, the early stages of this protein assembly process can be observed in HSV-1-infected cells [7], [40]. These assemblies, or prereplicative sites, contain ICP8, the helicase/primase complex (UL8/UL5/UL52), and the origin binding protein, UL9 [40], as well as the cellular proteins ATR/ATRIP and RPA [7]. The observation that ICP8 and UL8 are necessary to inhibit ATR signaling led us to examine whether these proteins are sufficient to inhibit ATR signaling, and whether their ability to reorganize the nucleus is also required for inhibition of ATR signaling. Structures that resemble replication compartments are detected in cells transfected with plasmids expressing the seven replication proteins [24], [42]. Foci that resemble prereplicative sites can also be reconstituted in cells transfected with plasmids expressing subsets of the replication proteins. Figure S2 shows that as previously described [23], [24], when expressed alone either ICP8 or the helicase/primase complex localize in a nuclear diffuse staining pattern. On the other hand, transfection of cells with plasmids expressing ICP8 and helicase/primase results in the formation of punctate nuclear structures that contain all four proteins and resemble prereplicative sites [24], [40]. The formation of the four-protein complex is consistent with the observation that ICP8 colocalizes with the helicase/primase complex by immunofluorescence and that the four proteins directly interact in a UL8 dependent fashion [23], [24], [31]. The four-protein complex forms efficiently in greater than 75% of transfected cells. When ICP8 is expressed with UL8 in the absence of UL5 and UL52, punctate structures can be detected, which we have termed two-protein complexes (Fig. S2); however, formation of these structures is less efficient than formation of the four-protein complex, forming in less than 10% of transfected cells.

**Figure 3:**
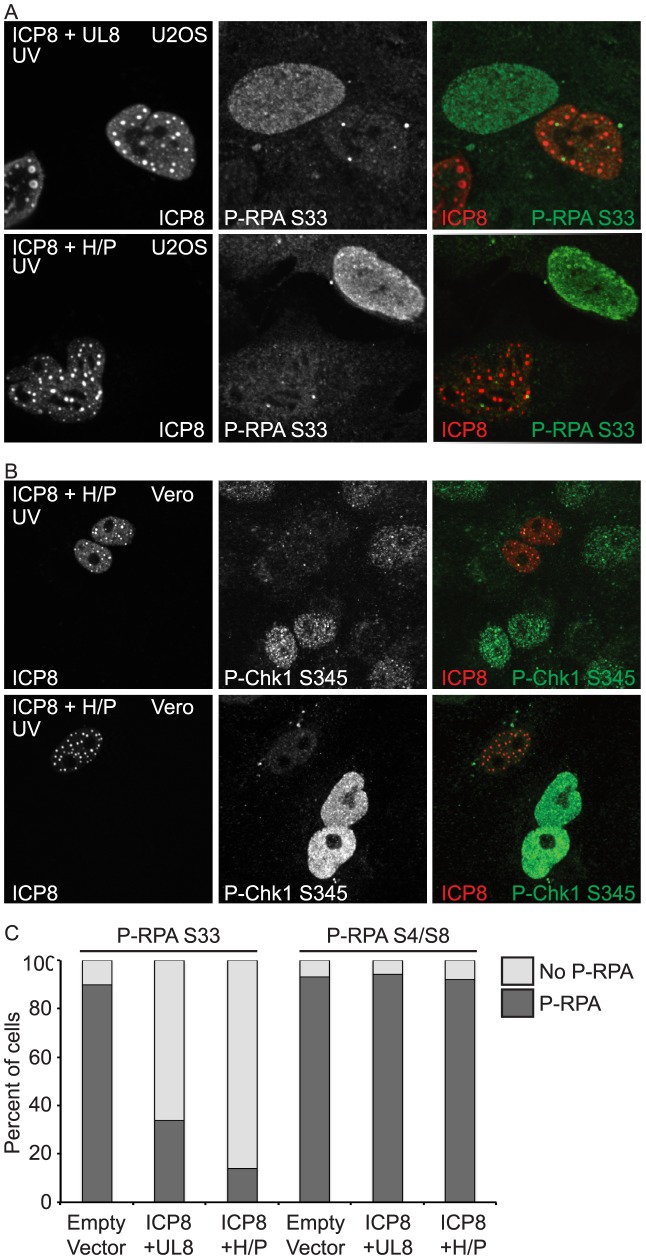
ICP8 and UL8 are sufficient to inhibit ATR signaling. (A) U2OS or (B) Vero cells were transfected with ICP8, UL8, UL5, and UL52 (ICP8+H/P) or ICP8 and UL8 alone and then damaged with UV. Cells were fixed at 1 hour post damage and prepared for immunofluorescence as described in the materials and methods. (C) Cells were treated as in A and stained for either P-RPA-S33 or P-RPA-S4/S8. At least 100 cells were counted between two independent experiments.

To determine whether ICP8 and UL8 are sufficient to inhibit ATR signaling, cells expressing the two-protein complex were damaged with UV and monitored for ATR activation by immunofluorescence. Untransfected cells exhibited phosphorylated RPA-S33 in response to UV, while cells expressing the two-protein complex did not (Fig. 3A). This result suggests that ICP8 and UL8 are necessary and sufficient to disable ATR signaling even in the presence of DNA damage that would normally activate ATR. Cells expressing the four-protein complex were also able to inhibit ATR signaling to RPA-S33 and Chk1 in response to UV (Fig. 3A and B). We further quantified this reduction in phosphorylated RPA S33 by counting cells expressing the two- or four-protein complex and scoring them for presence or absence of phosphorylated RPA (Fig. 3C). In UV-treated cells expressing an empty vector greater than 90% of the cells exhibited phosphorylated RPA S33. In contrast, UV-treated cells expressing the two- or four-protein complex exhibited 33% and 14% of cells with phosphorylated RPA S33, respectively. These data suggest that the two- and four-protein complexes are efficient inhibitors of ATR signaling.

In addition to the ATR specific phosphorylation on RPA S33 after UV damage, RPA is also phosphorylated by DNA-PK on S4/S8 [13], [43]. To test whether this inhibition was specific for ATR, we treated cells expressing the four-protein complex with UV and looked at phosphorylation of RPA S4/S8. We observed no difference in RPA S4/S8 phosphorylation between cells expressing an empty vector and cells expressing the two- or four-protein complex (Fig. 3C), indicating that neither complex can inhibit DNA-PK signaling. Thus, the two- and four-protein complexes are specific for inhibition of ATR signaling.

As a control we also verified that expression of ICP8 and helicase/primase did not alter the cell cycle profile of these cells (Fig. S3); therefore, the inhibition of ATR signaling is not due to a decreased number of cells in S-phase. Consistent with the observation that the four-protein complex forms more efficiently than the two-protein complex, the four-protein complex is more efficient than the two-protein complex at inhibiting ATR signaling (Fig. 3C). Furthermore, UL5/UL52 are unlikely to inhibit ATR signaling on their own since these proteins do not localize to the nucleus in cells that do not express UL8 [26]–[28], nor do they associate with ICP8 in the absence of UL8 [31]. Thus, we chose to focus the rest of our studies on the four-protein complex.

### UL8 mutants that do not support DNA replication still inhibit ATR signaling

**Figure 4:**
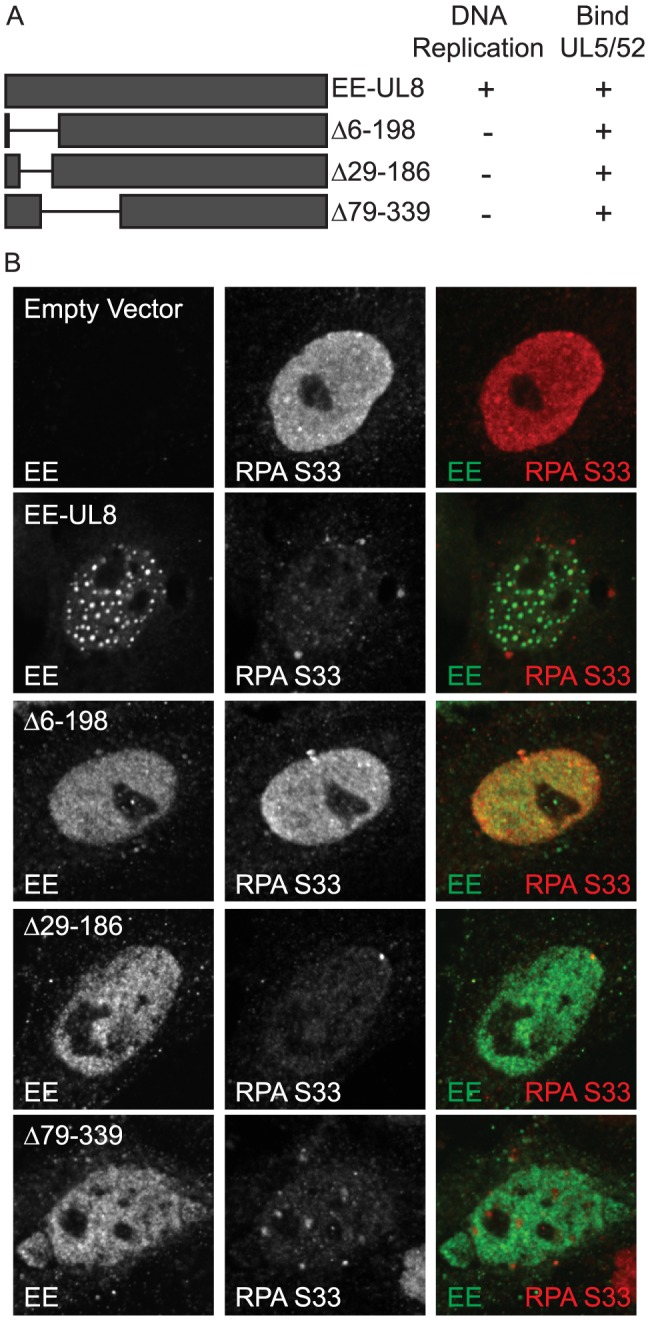
UL8 mutants that do not support DNA replication still inhibit ATR signaling. (A) Schematic of UL8 mutants used in this study. (B) Vero cells were transfected with ICP8, UL5, UL5, and the indicated UL8 mutants and then damaged with UV. Cells were fixed at 1 hour post damage and prepared for immunofluorescence as described in the materials and methods.

To date, no enzymatic function has been assigned to UL8, and it is believed to function as a scaffolding protein to link the helicase/primase complex to other replication factors [28], [31]–[36]. We have previously described a functional EE-epitope tagged version of UL8 (EE-UL8) [44]. Three internal deletion mutants (Δ6–198, Δ29–186, and Δ79–339) were generated in EE-UL8 (Fig. 4A). All three mutants are able to express stable protein that can interact with UL5 and UL52; however, they are unable to support origin-dependent DNA replication and four-protein complex formation (Figure S4). We tested the ability of these mutants to inhibit ATR activation. Vero cells were transfected with ICP8, UL5, UL52, and the indicated EE-UL8 mutant and then damaged with UV. EE-UL8, Δ29–186, and Δ79–339 were able to prevent ATR-dependent RPA S33 phosphorylation while Δ6–198 was not (Fig. 4B). These data confirm that DNA replication is dispensable for inhibiting ATR signaling. Furthermore, the inability of Δ6–198 to inhibit ATR signaling suggests that residues at the N-terminus of UL8 (between 6 and 29) may be required to inhibit ATR signaling. Since Δ29–186 and Δ79–339 do not form the four-protein complex but are still able to inhibit ATR, this suggests that the formation of the four-protein complex is not strictly required for this function.

### The four-protein complex localizes to sites of DNA damage

**Figure 5:**
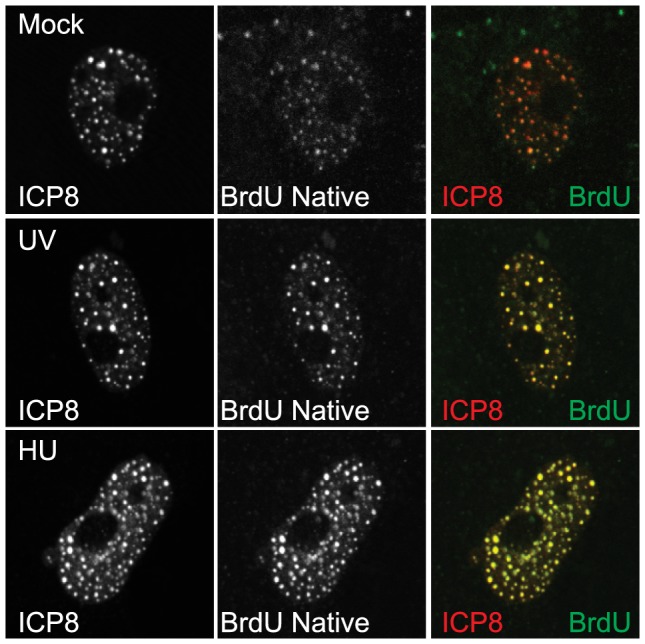
The four-protein complex localizes to sites of DNA damage. Vero cells were transfected with ICP8, UL8, UL5, and UL52 and BrdU was added at the time of transfection. Cells were treated with UV or HU at 24

Since ICP8 and helicase/primase are able to inhibit ATR signaling, we next asked whether they could localize to sites of DNA damage. Cells were transfected with plasmids encoding ICP8 and the helicase/primase complex, and BrdU was added to the media at the time of transfection to detect sites of cellular DNA replication. Cells were either left undamaged or damaged with UV or HU, and then analyzed for ICP8 and BrdU by immunofluorescence under non-denaturing conditions. Without the denaturation step, BrdU antibodies only detect BrdU in ssDNA. Figure 5 shows that ICP8 colocalizes with BrdU in both undamaged and damaged cells suggesting that the four-protein complex is present at ssDNA regions in undamaged cells and sites of DNA damage in the presence of UV or HU. Although ICP8 colocalizes with BrdU in both damaged and undamaged cells, BrdU staining is much brighter in damaged cells reflecting the increased amount of ssDNA known to be present at sites of DNA damage as a result of helicase and polymerase uncoupling [45]. The observation that ICP8 and helicase/primase localize to sites of ssDNA in undamaged and damaged cells suggests that the four-protein complex may also recognize and be recruited to sites of endogenous DNA damage in transfected cells. Consistent with this notion, the four-protein complex also colocalizes with Rad51 and γH2AX, known markers of DNA damage (Fig. S5).

### Rad9 and TopBP1 are excluded from the four-protein complex to prevent ATR signaling

**Figure 6:**
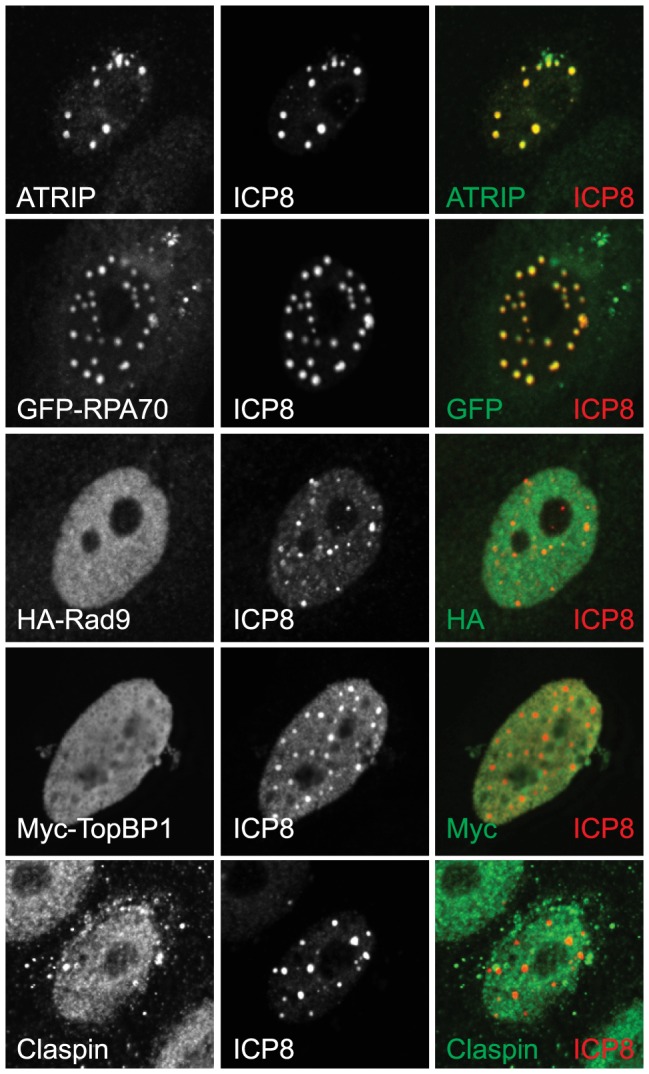
Essential ATR pathway proteins are excluded from the four-protein complex. Vero cells were transfected with ICP8, UL8, UL5, and UL52 and damaged with UV and allowed to recover for 1-RPA70 or Myc-TopBP1 were cotransfected with the viral proteins. To follow HA-Rad9 Vero cells stably expressing HA-Rad9 were transfected with the viral proteins prior to UV irradiation.

In order to determine whether cellular ATR pathway proteins such as ATR/ATRIP and RPA are recruited to the four-protein complex after damage, cells expressing ICP8 and helicase/primase were damaged with UV, fixed and analyzed by immunofluorescence. ATRIP and GFP-RPA70 were both strongly recruited to the four-protein complex and precisely colocalized with ICP8 (Fig. 6), consistent with our previous observation that endogenous RPA32 is recruited to the four-protein complex in 91% of cells expressing the four-protein complex [17]. Interestingly, we found that neither tagged nor endogenous Rad9, TopBP1, or Claspin were recruited to the four-protein complex (Fig. 6 and S5). For instance, 55 of 55 cells expressing the four-protein complex exhibited diffuse Rad9. As noted previously, the four-protein complex resembles prereplicative sites generated during infection in the presence of helicase/primase inhibitors. The helicase/primase inhibitor used in this study inhibits helicase/primase activity and DNA synthesis but does not alter the expression levels of helicase/primase and does not alter their localization with ICP8 in prereplicative sites. Interestingly, ATR/ATRIP and RPA are recruited to prereplicative sites, and Rad9 is not ([7] and Figure S6). Exclusion of these essential ATR signaling co-factors from prereplicative sites and from the four-protein complex provides an explanation for the lack of ATR signaling in cells expressing ICP8 and helicase/primase.

**Figure 7:**
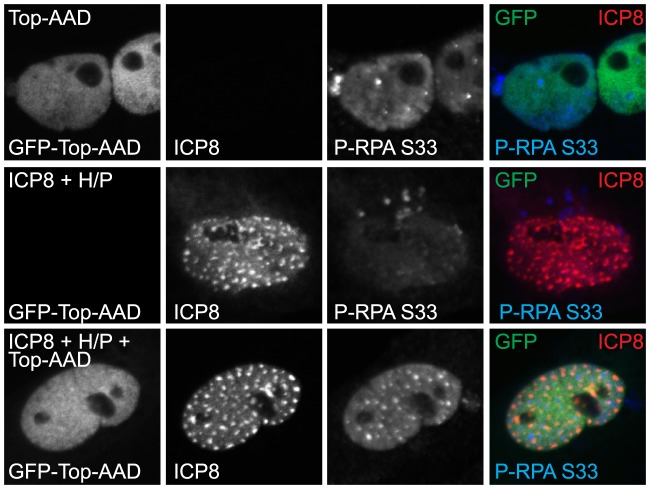
ATR can be activated in cells expressing the four-protein complex and the ATR Activation Domain of TopBP1. Vero cells were transfected with GFP-TopBP1-AAD alone or in combination with ICP8, UL8, UL5, and UL52 (ICP8+H/P). Cells were fixed at 18 hours post transfection and prepared for immunofluorescence as described in the materials and methods.

To further test the hypothesis that lack of 9-1-1 recruitment functionally inactivates ATR by preventing ATR-interaction with TopBP1, we took advantage of a previously described TopBP1 mutant that overcomes the need for 9-1-1 in ATR activation. The over-expression of the ATR activation domain (AAD) of TopBP1 (amino acids 978–1286) specifically activates ATR signaling [12], [18], [46], [47]. TopBP1-AAD lacks the 9-1-1 interacting domain but is still able to bind and activate ATR. Thus, if the four-protein complex inhibits ATR by preventing 9-1-1-mediated recruitment of TopBP1, then expressing TopBP1-AAD should restore ATR signaling. This is indeed the case, as TopBP1-AAD stimulated ATR signaling when transfected alone and when transfected with the four-protein complex (Fig. 7). This experiment also indicates that ATR is still functional in cells expressing the four-protein complex. Thus, the inactivation of ATR signaling in HSV-infected cells is not due to inactivation by post-translational modifications or dephosphorylation of ATR substrates. Therefore, the mechanism by which ATR signaling is disabled in HSV-infected cells involves the lack of 9-1-1recruitment to sites of damage.

## Discussion

In this paper we report that HSV-1 specifically disables ATR signaling by preventing the essential ATR cofactors Rad9 and TopBP1 from accessing sites of DNA damage. We report that the intra-nuclear complexes formed by over-expression of ICP8 and UL8 alone (two-protein complex) or ICP8 and helicase/primase (four-protein complex) are necessary and sufficient to disable ATR signaling. The four-protein complex is recruited to sites of DNA damage and inhibits ATR signaling to RPA and Chk1. The four-protein complex contains ATR/ATRIP and RPA but does not contain other essential ATR pathway proteins Rad9, TopBP1, and Claspin.

### Recruitment of viral and cellular proteins to sites of ssDNA

**Figure 8:**
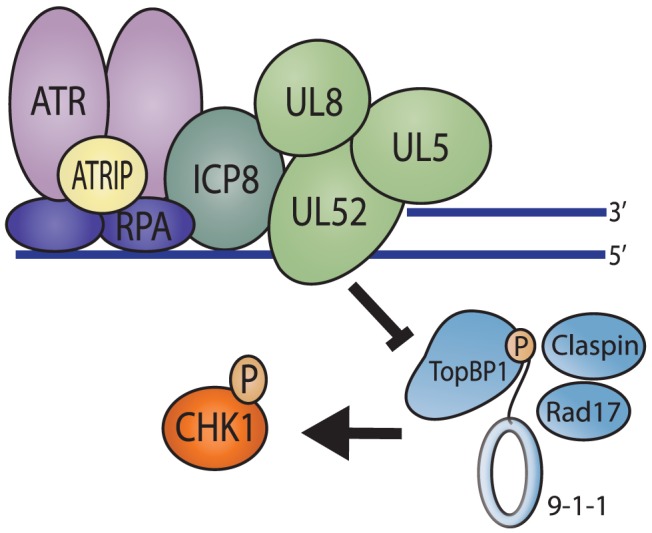
Model of DNA repair proteins at sites of DNA damage in the presence of the 4-protein complex. In the presence of ICP8 and helicase/primase, RPA can still coat ssDNA at sites of damage and recruit ATR/ATRIP. However, these four-proteins bind to the ss/dsDNA junction that would normally serve as the loading platform for the 9-1-1 complex and exclude it from binding the DNA. This serves to prevent all of the downstream proteins from being recruited to sites of DNA damage and effectively inhibits ATR signaling.

Several of the proteins described in this study are known to participate in protein-protein as well as protein-DNA interactions. UV- or HU-induced damage is expected to result in a DNA structure containing ssDNA adjacent to dsDNA. This type of structure is also present on the lagging strand during DNA synthesis. We have previously shown that the helicase/primase complex has a higher affinity for forked DNA or dsDNA with a ssDNA overhang than for ssDNA or dsDNA and that dimer or higher-order complexes of helicase/primase could form on forked DNA substrates [48], [49]. Thus, HSV-1 helicase/primase is known to bind DNA substrates that have similar structures to those recognized by the 9-1-1 clamp in damaged DNA or at stalled replication forks. We suggest that when viral DNA synthesis stalls, helicase/primase binds at ss/dsDNA junctions on the lagging strand and effectively prevents 9-1-1 from loading. Consistent with the proposed model, we also observe the four-protein complex at sites of replication/repair in undamaged cells. Since TopBP1 is generally recruited to sites of DNA damage by interacting with the C-terminal tail of 9-1-1, the failure to load 9-1-1 would preclude TopBP1 binding. In support of this model, we failed to detect 9-1-1 or TopBP1 at sites of damage in cells transfected with ICP8 and helicase/primase. The inability to recruit TopBP1, the ATR kinase activator, explains the lack of detectable ATR signaling to RPA and Chk1. As a further test of the model, we expressed a mutant form of TopBP1 that overcomes the need for 9-1-1 recruitment and restored ATR signaling. Together these observations provide a compelling model to explain the inhibition of ATR signaling by HSV-1. The model shown in Figure 8 is consistent with the available data; however, validation will require additional experimentation including more direct demonstration of competition between helicase/primase and 9-1-1 for loading onto the ssDNA-dsDNA junction *in vitro*. Mammalian 9-1-1 loading onto ss/dsDNA junctions has not been reconstituted *in vitro*, so it is not immediately possible to directly test this part of the model *in vitro*. Thus, other mechanisms cannot be excluded at this time.

The current study was initiated to determine how ATR signaling is disabled in HSV-1-infected cells even though ATM signaling is activated by viral DNA replication. ATR signaling is believed to be important for the stabilization of stalled replication forks. The prevention of ATR signaling during infection may predispose viral replication forks to collapse which in turn may lead to DSB formation, ATM activation and homology directed repair. Since we and others have suggested that HSV-1 utilizes recombination-mediated DNA synthesis [22], [50] it is possible that inactivation of ATR signaling is beneficial for the virus. This interpretation is also consistent with our previously published observation that constitutively activated ATR inhibits HSV-1 replication-dependent recombination [18], possibly through stabilization of a stalled fork. It is also important to point out that while TopBP1 and Rad9 are excluded from the four-protein complex, they are recruited to replication compartments [18]. Although we have shown that they do not participate in ATR signaling in infected cells, TopBP1 and Rad9 are known to interact with several other DNA repair and replication proteins that could recruit them to replication compartments. For example, TopBP1 is present in cellular DNA replication complexes and also interacts with ATM after double strand breaks. Likewise, Rad9 binds MLH1, a protein known to bind viral DNA, and is also recruited to DNA double strand breaks by an interaction with Mre11 and CtIP[51]–[58]. Thus, these proteins could be recruited by distinct mechanisms that do not result in ATR activation.

### Implications for inhibition of ATR signaling

ATR is an essential cellular protein [59], [60]; however, unlike its related kinase, ATM, the regulation of ATR is poorly understood. Also unlike ATM, there is no consensus regarding a reliable phosphorylation mark that is indicative of ATR activation [61], [62], and ATR specific inhibitors are just beginning to emerge [63]–[65]. Many DNA viruses including herpesviruses, adenoviruses and autonomous and non-autonomous parvoviruses disable ATR signaling. Reports from the Weitzman and Turnell labs show that Adenoviruses 5 and 12 disable ATR signaling by E4-mediated degradation of MRN and TopBP1 respectively [66], [67] This paper presents the first example of a DNA virus that disables cellular ATR signaling by preventing cellular DNA repair proteins from accessing the DNA. This report is also the first to suggest that viral DNA replication machinery can alter the cellular recognition and signaling pathways for DNA damage in infected cells. To date, all other examples of viral manipulation of the DNA damage response have relied on viral proteins such as E4orf3 and E4orf6 in adenovirus or ICP0 in HSV-1 that specifically recognize and degrade substrates irrespective of damage.

In this study we have shown that HSV-1 replication proteins can prevent Rad9 and TopBP1 from accessing sites of DNA damage and prevent ATR activation. The use of viruses to target cancer cells (oncolytic virotherapy) either alone or in combination with conventional chemotherapy may provide a novel way to inhibit ATR signaling. For example, ATM- and p53-deficient tumor cells are very sensitive to ATR inhibition [63], and the combination of oncolytic HSV-1 with conventional chemotherapy has resulted in decreased tumor volume and improved long-term survival in animal models of Glioblastoma multiforme [68], [69]. We suggest that the benefits of combining oncolytic HSV-1 with conventional chemotherapy are due to the ability of HSV-1 to specifically disable ATR signaling and thus sensitize cancer cells to DNA damaging agents.

## Materials and Methods

### Cells and reagents

Vero, HeLa, and U2OS cells were purchased from the American Type Culture Collection (ATCC) and maintained as previously described [52]. The Vero cell line stably expressing HA-Rad9 was previously described [18]. The helicase/primase inhibitor BAY 57-1293 (N-(5-(aminiosulfonyl)-4-methyl-1,3-thiazol-2-yl)-N-methyl-2-(4-(2-pyridinyl)phenyl)acetamide) was obtained from Gerald Kleymann (Bayer Pharmaceuticals; Wuppertal, Germany) [70] and used at a concentration of 100 µM as described [7], [40]. The polymerase inhibitor phosphonoacetic acid (PAA) was purchased from Sigma and used at a concentration of 400 µg/mL as previously described [17]. In all DNA damage experiments hydroxyurea was purchase from Sigma and used at a concentration of 3 mM or cells were damaged with 50 J/m^2^ UV.

### Viruses

The KOS strain was used as wild type HSV-1 and all mutant viruses used in this study are derived from KOS. The following viruses were previously described: ΔICP0 (0β) [71], ΔICP4 (d120) [72], ΔICP22 (d22lacZ) [73], ΔICP27 (d27-1) [74], ΔICP4/ICP27 (d92) [75], ΔICP4/22/27/47 (d106) [76], ΔICP8 (HD2) [77], ΔUL5 (hr99) [78], ΔUL8 (hr80) [79], ΔUL52 (hr114) [80], and ΔUL30 (hp66) [81].

### Plasmids and transfections

Proteins expressed from CMV promoters were previously described, ICP8 (pCM-DBP), UL8 (pCM-UL8), UL5 (pCM-UL5b), UL52 (pCMV-UL52), GFP-RPA70 (pEGFP-RPA70), GFP-TopBP1-AAD, and Myc-TopBP1 [18], [24], [46], [82], [83]. Cells were transfected with Lipofectamine PLUS (Invitrogen) according to the manufacturer's suggested protocol.

### Immunofluorescence

IF analysis was performed as described [7], [40], [52]. Briefly, cells adhered to glass coverslips were washed with PBS, fixed with 4% paraformaldehyde, and permeabilized with 1% Triton X-100. Cells were blocked in 3% normal goat serum and reacted with antibodies as indicated. Staining for BrdU was done as previously described with the omission of a HCl wash to denature DNA [24]. Primary antibodies include polyclonal rabbit anti-ATRIP (rATRIP Upstate) (1∶200; Upstate), monoclonal mouse anti-ICP8 (1∶200; Abcam), polyclonal rabbit anti-ICP8 367 [84], monoclonal rabbit anti-phospho-Chk1 S345 (1∶200; Cell Signaling), polyclonal rabbit anti-phospho-RPA S33 (1∶200; Bethyl), monoclonal rat anti-BrdU (1∶100; Genetex), polyclonal rabbit anti-Claspin (1∶200; Bethyl), monoclonal mouse anti-Myc (9B11) (1∶200; Cell Signaling), monoclonal rat anti-HA (1∶200; Roche), and polyclonal rabbit anti-HA (1∶200;Clontech). AlexaFluor secondary antibodies (1∶200; Molecular Probes) were used with fluorophores excitable at wavelengths of 488, 594, or 647. Images were captured using a Zeiss LSM 510 confocal NLO microscope equipped with argon and HeNe lasers and a Zeiss 63× objective lens (numerical aperture, 1.4). Images were processed and arranged using Adobe Photoshop CS3 and Illustrator CS3.

### Western blot analysis

Cells in 35 mm dishes were lysed in 2× SDS sample buffer (4% SDS, 20% glycerol, 100 mM Tris pH 6.8, 100 mM DTT, 10% β-Mercaptoethanol, 1 mM sodium orthovanadate, 10 mM NaF, 1× protease inhibitor cocktail (Roche), and 0.1% bromophenol blue) and boiled for 5 minutes. Proteins were resolved by SDS-PAGE and transferred to PVDF membranes. Membranes were blocked for 1 hour in 5% non-fat dry milk or 2% BSA dissolved in TBST. Primary antibodies were diluted in blocking solution and incubated overnight at 4°C. Primary antibodies used include polyclonal rabbit anti-ATRIP 403 (rATRIP 403) (1∶3,000) [59], monoclonal mouse anti-ICP4 (1∶10,000; US Biologics), monoclonal mouse anti-β-actin (1∶15,000; Sigma), polyclonal goat anti-ATR N19 (1∶1,000; Santa Cruz), monoclonal mouse anti-Chk1 (1∶1,000; Santa Cruz), monoclonal mouse anti-HA (F7) (1∶3,000; Santa Cruz), monoclonal mouse anti-RPA32 (9H8) (1∶1,000; Genetex), monoclonal rabbit anti-phospho-Chk1 S345 (1∶5,000; Cell Signaling), polyclonal rabbit anti-phospho-RPA S33 (1∶3,000; Bethyl), and polyclonal rabbit anti-phospho-RPA S4/S8 (1∶3,000; Bethyl). Polyclonal rabbit antisera to UL8 (R248) and UL52 (R2403) were provided by Mark Challberg.

## Supporting Information

Figure S1 **Resolution of DNA damage during HSV-1 infection.** Vero cells were treated with 3 mM HU for 24 hours. HU was then washed out and cells were either mock infected or infected with HSV-1 at an MOI of 10 PFU/cell and harvested at the indicated time points for Western blot analysis. Time −1 hr represents the time virus was initially added to cells and time 0 hr represents the end of the one hour virus adsorption period.
(EPS)

Figure S2 **Nuclear structures formed in the presence of ICP8 and helicase/primase.** Vero cells were transfected with the indicated combinations of ICP8 and helicase/primase (EE-UL8/UL5/UL52) and fixed at 18 hours post transfection. Immunofluorescence was performed with antibodies directed against ICP8 and EE.
(EPS)

Figure S3 **Expression of ICP8 and helicase/primase does not alter the cell cycle profile of cells.** Vero cells were transfected with the indicated combinations of ICP8 and helicase/primase. Samples were fixed in 70% EtOH 24, 48 and 72 hours post transfection and stained for 30 min with propidium iodide (PI) staining solution (PBS, 0.1% Trition-X, 0.2 mg/ml Rnase A and 0.02 mg/ml PI). Cell Cycle distribution was measured using a Becton Dickinson LSR II flow cytometer and analyzed using ModFit LT software. At least 20,000 G1 events were collected for each sample.
(EPS)

Figure S4 **UL8 mutants interact with UL5 and UL52 but do not support DNA replication or four-protein complex formation.** (A) Schematic of the UL8 mutants used in this study. (B) Sf9 cells were infected with baculoviruses expressing UL5, UL52, and the indicated UL8 constructs. Cells were labeled with ^35^S-Met 10 hours before harvesting. Immunprecipitations were performed using the indicated antibodies, separated by SDS-PAGE, and exposed to film. (C) The indicated UL8 plasmids were transfected into Vero cells along with pOriS and then cells were infected with the UL8-null virus. Plasmids were recovered and digested with EcoR1 and Dpn1 as indicated and a southern blot was performed using a pUC118 probe. (D) Vero cells were transfected with ICP8, UL5, UL52, and the indicated UL8 mutants. Cells were fixed at 18 hours post transfection and stained for EE and ICP8.
(EPS)

Figure S5 **Rad51 and γH2AX are recruited to the four-protein complex.** Vero cells were treated as in Figure 6A and prepared for immunofluorescence as described in the materials and methods.
(EPS)

Figure S6 **Rad9 is not recruited to the four-protein complex or prereplicative sites.** Vero cells stably expressing HA-Rad9 were either transfected with ICP8, UL8, UL5, and UL52 (ICP8+H/P), infected with HSV-1 at an MOI of 2 PFU/cell in the presence (HSV-1+BAY) or absence (HSV-1) of the helicase/primase inhibitor BAY 57–1293. Cells were fixed at 6 hours post infection and stained for ICP8 and HA.
(EPS)
